# HER2 and TOP2A in high-risk early breast cancer patients treated with adjuvant epirubicin-based dose-dense sequential chemotherapy

**DOI:** 10.1186/1479-5876-10-10

**Published:** 2012-01-12

**Authors:** George Fountzilas, Christos Valavanis, Vassiliki Kotoula, Anastasia G Eleftheraki, Konstantine T Kalogeras, Olympia Tzaida, Anna Batistatou, Ralf Kronenwett, Ralph M Wirtz, Mattheos Bobos, Eleni Timotheadou, Nikolaos Soupos, George Pentheroudakis, Helen Gogas, Dimitrios Vlachodimitropoulos, Genovefa Polychronidou, Gerasimos Aravantinos, Angelos Koutras, Christos Christodoulou, Dimitrios Pectasides, Petroula Arapantoni

**Affiliations:** 1 Department of Medical Oncology, Papageorgiou Hospital, Aristotle University of Thessaloniki School of Medicine, Thessaloniki, Greece; 2 Department of Pathology, Metaxas Cancer Hospital, Piraeus, Greece; 3 Department of Pathology, Aristotle University of Thessaloniki School of Medicine, Thessaloniki, Greece; 4 Section of Biostatistics, Hellenic Cooperative Oncology Group, Data Office, Athens, Greece; 5 Translational Research Section, Hellenic Cooperative Oncology Group, Data Office, Athens, Greece; 6 Department of Pathology, Ioannina University Hospital, Ioannina, Greece; 7 Siemens Healthcare Diagnostics, Cologne, Germany; 8 Laboratory of Molecular Oncology, Hellenic Foundation for Cancer Research, Aristotle University of Thessaloniki School of Medicine, Thessaloniki, Greece; 9 Department of Clinical Therapeutics, Alexandra Hospital, University of Athens School of Medicine, Athens, Greece; 10 Department of Medical Oncology, Ioannina University Hospital, Ioannina, Greece; 11 First Department of Medicine, Laiko General Hospital, University of Athens, Medical School, Athens, Greece; 12 Laboratory of Pathology, Evgenidion Hospital, University of Athens Medical School, Greece; 13 Third Department of Medical Oncology, Agii Anargiri Cancer Hospital, Athens, Greece; 14 Department of Medicine, Division of Oncology, University Hospital, University of Patras Medical School, Patras, Greece; 15 Second Department of Medical Oncology, Metropolitan Hospital, Piraeus, Greece; 16 Oncology Section, Second Department of Internal Medicine, Hippokration Hospital, Athens, Greece; 17 Current: Sividon Diagnostics GmbH, Nattermann Allee 1, D-50829 Cologne, Germany; 18 Current: Stratifyer Molecular Pathology GmbH, Werthmannstrasse 1, D-50935 Cologne, Germany

**Keywords:** HER2, TOP2A, gene amplification, CISH, mRNA expression, early breast cancer, randomized study, anthracyclines, taxanes

## Abstract

**Background:**

HER2 and TOP2A parameters (gene status, mRNA and protein expression) have individually been associated with the outcome of patients treated with anthracyclines. The aim of this study was to comprehensively evaluate the prognostic/predictive significance of the above parameters in early, high-risk breast cancer patients treated with epirubicin-based, dose-dense sequential adjuvant chemotherapy.

**Methods:**

In a series of 352 breast carcinoma tissues from patients that had been post-operatively treated with epirubicin-CMF with or without paclitaxel, we assessed HER2 and TOP2A gene status (chromogenic in situ hybridization), mRNA expression (quantitative reverse transcription PCR), as well as HER2 and TopoIIa protein expression (immunohistochemistry).

**Results:**

HER2 and TOP2A amplification did not share the same effects on their downstream molecules, with consistent patterns observed in HER2 mRNA and protein expression according to HER2 amplification (all parameters strongly inter-related, p values < 0.001), but inconsistent patterns in the case of TOP2A. TOP2A gene amplification (7% of all cases) was not related to TOP2A mRNA and TopoIIa protein expression, while TOP2A mRNA and TopoIIa protein were strongly related to each other (p < 0.001). Hence, TOP2A amplified tumors did not correspond to tumors with high TOP2A mRNA or TopoIIa protein expression, while the latter were characterized by high Ki67 scores (p = 0.003 and p < 0.001, respectively). Multivariate analysis adjusted for nodal involvement, hormone receptor status, Ki67 score and HER2/TOP2A parameters revealed HER2/TOP2A co-amplification (21.2% of HER2 amplified tumors) as an independent favorable prognostic factor for DFS (HR = 0.13, 95% CI: 0.02-0.96, p = 0.046); in contrast, increased HER2/TOP2A mRNA co-expression was identified as an independent adverse prognostic factor for both DFS (HR = 2.41, 95% CI: 1.31-4.42, p = 0.005) and OS (HR = 2.83, 95% CI: 1.42-5.63, p = 0.003), while high TOP2A mRNA expression was an independent adverse prognostic factor for OS (HR = 2.06, 95% CI: 1.23-3.46, p = 0.006). None of the parameters tested was associated with response to paclitaxel.

**Conclusions:**

This study confirms the favorable prognostic value of HER2/TOP2A co-amplification and the adverse prognostic value of high TOP2A mRNA expression extending it to the adjuvant treatment setting in early high-risk breast cancer. The strong adverse prognostic impact of high HER2/TOP2A mRNA co-expression needs further validation in studies designed to evaluate markers predictive for anthracyclines.

**Trial registration:**

Australian New Zealand Clinical Trials Registry ACTRN12611000506998.

## Introduction

Adjuvant chemotherapy is known to prolong disease-free survival (DFS) and overall survival (OS) in patients with early-stage breast cancer (EBCTCG [1]). Moreover, anthracyclines and taxanes are currently considered to be essential drugs in this setting [2]. However, the indiscriminate administration of these drugs and especially of anthracyclines results in late life-threatening toxicities, such as congestive heart failure and acute leukemia or myelodysplasia, in 0.3 to 3% of the patients [3,4]. The above potential risks point out the importance of the identification of biological markers, which would separate patient subgroups with favorable prognosis and thus spare them the potential risks of such toxic treatment.

HER2 gene amplification and/or protein overexpression has been identified in approximately 20% of invasive breast cancer patients [5] and was shown to be associated with a worse prognosis [6]. HER2 gene amplification has been evaluated as a predictive factor for different cytotoxic drugs, including anthracyclines and paclitaxel [7,8]. Interestingly, most relevant studies and a meta-analysis [9] provided compelling evidence that the benefit from adjuvant anthracyclines is restricted to the HER2-positive subgroup of patients. Nevertheless, these results have been challenged by other investigators, especially in patients with metastatic disease [10,11]. Taken together, these findings raise the possibility that other than HER2 genes, located on chromosome 17, may be key regulators of anthracycline responsiveness [7].

One such gene is topoisomerase II alpha (TOP2A), which is located ~700 kb telomeric to HER2 and encodes a cell-cycle regulator. TOP2A is a key player in DNA replication and remodeling and, in the context of cytotoxic chemotherapy, a molecular target for anthracyclines and other chemotherapeutic agents [12,13]. TOP2A gene is co-amplified in 30%-40% of the tumors with HER2 gene amplification, while deletions are also frequently observed [14]. The puzzle on the possible prognostic/predictive role of TOP2A is complicated by studies suggesting that topoisomerase II alpha protein expression (TopoIIα), rather than TOP2A gene amplification, is predictive of anthracycline responsiveness in the adjuvant setting [15].

HER2 and TOP2A gene amplification testing is mostly performed by fluorescent in situ hybridization (FISH) or chromogenic in situ hybridization (CISH) [16]. However, neither of these two molecular techniques is characterized by high resolution for gene mapping and may easily miss gene-specific microdeletions identified by other approaches, such as PCR-based methods.

Based upon this information we performed a comprehensive evaluation of HER2 and TOP2A parameters in tumors from patients that had participated in the Hellenic Cooperative Oncology Group (HeCOG) phase III trial HE10/97 [17]. The objective of this trial had been the evaluation of the efficacy of epirubicin-based dose-dense sequential regimens with and without paclitaxel in patients with high-risk operable breast cancer. In the present study, we sought to investigate HER2 and TOP2A parameters at three different levels (gene status, mRNA expression and protein expression) in association with classic clinicopathological parameters, as well as to evaluate their impact on patient outcome. Clearly, since prognostic and predictive factors represent distinct means for assessing clinical outcome [18], and since all our patients had been treated with an anthracycline, it was possible to investigate for prognostic factors and not for factors predicting response to anthracyclines in the present setting. However, since paclitaxel had been added to the regimen in half of the cases, we also evaluated HER2 and TOP2A parameters as markers predictive for response to this agent.

## Patients and methods

### Patients and tissues

Formalin-fixed paraffin-embedded (FFPE) tumor tissue samples were retrospectively collected from 367 patients that had participated in a phase III trial (HE 10/97); in that trial, 595 eligible high-risk breast cancer patients were randomized to receive postoperative dose-dense sequential chemotherapy with 3 cycles of epirubicin 110 mg/m^2^, followed by 3 cycles of paclitaxel (Taxol™, Bristol-Myers Squibb, Princeton, NJ, USA) 250 mg/m^2^, followed by 3 cycles of intensified CMF (cyclophosphamide 840 mg/m^2^, methotrexate 57 mg/m^2^, fluorouracil 840 mg/m^2^) (E-T-CMF, experimental arm) or four cycles of epirubicin followed by 4 cycles of CMF (E-CMF, control arm), at the same doses as in the E-T-CMF arm. All cycles were given every 2 weeks with G-CSF support. The results of the HE10/97 have previously been published [17].

The clinical protocol and the related translational research studies were approved by the HeCOG Protocol Review Committee and by the Institutional Review Boards of ''Kyanous Stavros'' and ''AHEPA'' Hospitals and were carried out in compliance with the Helsinki Declaration. The trial was included in the Australian New Zealand Clinical Trials Registry (ANZCTR) and allocated the following Registration Number: ACTRN12611000506998. Upon participation in the trial, all patients provided a written informed consent for molecular studies of their tumor specimen.

**Figure 1:**
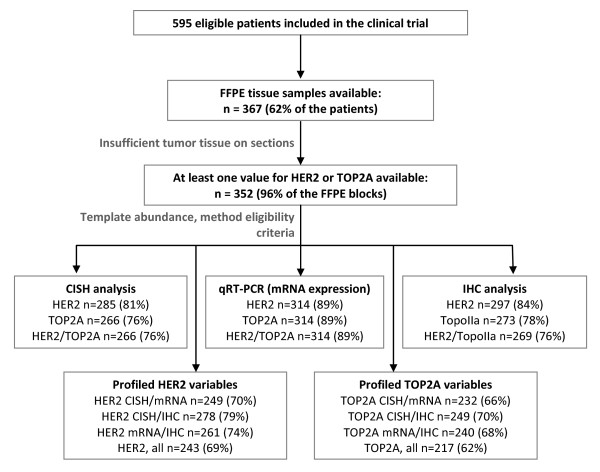
**REMARK diagram detailing FFPE tissue and sample availability in the present study for the application of different analytical techniques for the determination of HER2 and TOP2A gene status, mRNA and protein expression**. The rates in parentheses for CISH, qRT-PCR and IHC were calculated against the number of tissue samples with at least one HER2 or TOP2A value available (n = 352).

Paraffin sections were histologically evaluated for tumor adequacy and for tumor cell content. Where possible, tissue microarray (TMA) blocks were constructed as previously described [19-21] with a manual arrayer (Model I, Beecher Instruments, Sun Prairie, WI), including two 1.5 mm cores per tumor and multiple neoplastic and non-neoplastic tissue samples as controls for slide-based assays. A REMARK diagram for the translational research studies is provided in Figure 1.

### Chromogenic in situ hybridization (CISH)

CISH was performed on all available tissue sections (TMA or whole sections) by using SPoT-Light probes from ZYMED™ (Invitrogen, Carlsbad, CA) for the HER2 gene, TOP2A gene and Chromosome 17 centromeric region. The method was performed as described by the manufacturer, with slight modifications, on three separate slides, one for each probe. The specimens were classified, according to the number of gene hybridization signals per nucleus in more than 50% of cancer cells of the infiltrative tumor component, in one of five CISH ranks: rank 1: monosomy, if one signal per nucleus was counted using the Chromosome 17 centromeric probe; rank 2: diploid, with two signals per nucleus; rank 3: polysomy, if tumor nuclei contained 3 to 5 signals using the Chromosome 17 centromeric probe or if a very low gene amplification with 3 to 5 gene copies per nucleus was identified in diploid Chromosome 17 neoplastic cells; rank 4: low gene amplification, if 6-10 signals or small clusters were counted per nucleus; rank 5: high gene amplification, if > 10 signals or large clusters were detected per nucleus. CISH was considered positive for amplification at ranks 4 and 5 [22,23].

### RNA extraction and mRNA investigations

RNA extraction was performed from whole or macrodissected 10 μm paraffin sections at the Molecular Research Laboratory of Siemens Healthcare Diagnostics (Cologne, Germany), by using a fully automated isolation method for total RNA, based on silica-coated magnetic beads (Versant Tissue Preparation Reagents, Siemens Healthcare Diagnostics, Tarrytown, NY) in combination with a liquid handling robot, as previously described in detail [24,25]. In brief, this type of dual nuclear acid extraction with silica coated magnetic beads involves an extra step of DNase I digestion ensuring the presence of pure RNA and the absence of DNA in the sample and yields molecular templates of adequate quality from FFPE sections for the assessment of gene expression with quantitative real-time PCR. The tumor cell content of the FFPE sections used for the RNA extraction was > 30% in practically all (97%) of the samples, and > 50% in the majority (76%) of the samples.

RNA samples were assessed by quantitative one-step reverse transcription-PCR (qRT-PCR) with FAM-TAMRA labelled Taqman assays for the relative expression of *ERBB2 *and *TOP2A genes. RPL37A expression *(ribosomal protein L37a) was assessed for sample normalization and for the evaluation of RNA content. Forward and reverse primers, probes, amplicon length, location according to the coding NCBI reference sequence, as well as percentage of PCR efficiency [E = 1^(10-slope)^] for each assay were as follows (data in the same order): for *ERBB2*, 5'-CCAGCCTTCGACAACCTCTATT-3', 5'-TGCCGTAGGTGTCCCTTTG-3', 5'-FAM-ACCAGGACCCACCAGAGCGGG-TAMRA-3', 87 bp, exon 27 (NM_004448.2 and all ERBB2 variants), 95.7%; for *TOP2A*, 5'- CATTGAAGACGCTTCGTTATGG-3', 5'- CCAGTTGTGATGGATAAAATTAATCAG-3', 5'-FAM-CAGATCAGGACCAAGATGGTTCCCACAT-TAMRA-3', 104 bp, exons 13-14 (NM_001067.3), 85.2%; for *RPL37A*, 5'-TGTGGTTCCTGCATGAAGACA-3', 5'-GTGACAGCGGAAGTGGTATTGTAC-3', 5'-FAM-TGGCTGGCGGTGCCTGGA-TAMRA-3', 65 bp, exons 3-4 (NM_000998.4), 86.0%. Reverse transcription real-time quantitative PCR was performed with the SuperScript^® ^III Platinum^® ^One-Step Quantitative RT-PCR System with ROX (Invitrogen, Karlsruhe, Germany) according to the manufacturer's instructions. Experiments were accomplished in an ABI PRISM^® ^7900HT system (Applied Biosystems, Darmstadt, Germany) with 30 min at 50°C and 2 min at 95°C followed by 40 cycles of 15 s at 95°C, and 30 s at 60°C, as previously described in detail [26]. Samples were run in triplicates and were considered eligible for analysis when *RPL37A *CT (cycle threshold) values were < 32 (triplicate mean values). Relative expression levels of the target genes were calculated as 40-dCT (i.e., 40-[CT_target_-CT_RPL37A_]) to yield positively correlated numbers and facilitate comparisons.

### Immunohistochemistry

Immunohistochemical methodology was employed for the assessment of classic breast cancer protein markers, i.e., estrogen receptor alpha (ER), progesterone receptor (PgR) and HER2, as well as of the proliferation marker Ki67 and topoisomerase II-alpha (TopoIIa). Immunohistochemical staining was performed according to standard protocols, with slight modifications, on serial 2.5 μm thick sections from the TMA blocks using the Bond Max™ (Leica Microsystems, Germany/Menarini Diagnostics, Athens, Greece) and i6000 (Biogenex, San Ramon, CA) autostainers. To assure optimal reactivity, immunostaining was applied within a period of 3-10 days after sectioning. Where possible, non-informative cases, as well cases not embedded on TMAs were re-cut from the original blocks and used for protein and gene analysis. Immunohistochemical evaluation was based on established or proposed criteria [23,27-29]. The methods employed for each marker (antibodies and processing conditions) as well as the evaluation criteria for each protein target are shown in Table 1.

**Table 1:** Proteins, source and dilution of antibodies, staining procedures and patterns and interpretation of results

Protein	Antibody [clone, source]	Ab dilution	Pretreatment/EU	IT	IHC Staining Detection System/Chromogen	Scoring System	Cut-off (%)	Staining Pattern	Reference
Ki67	MIB1 (1)	1:70	15'/EU2	20'	Polymer HRP/DAB	SQ	≥ 14%*	Nuclear	[28]
HER2	PL (1)	1:200	25'/EU1	30'	Polymer HRP/DAB	0-3	> 30%^#^	Membranous	[23]
TopoIIa	KiS1 (1)	1:200	15'/EU2	30'	EnVision/DAB	SQ	> 5%**	Nuclear	[27]
ER	6F11 (2)	1:70	20'/EU1	20'	Polymer HRP/DAB	H-Score	≥ 1%	Nuclear	[29]
PgR	1A6 (2)	1:70	20'/EU1	20'	Polymer HRP/DAB	H-Score	≥ 1%	Nuclear	[29]

Antigen Retrieval was done on a hot plate at 98°C.

DAB: 3,3'-Diaminobenzidine; EU: Epitope Unmasking; EU1: Citric acid, pH 6.0; EU2: Ethylenediaminetetraacetate, pH 8.8; HRP: Horseradish Peroxidase; IT: Incubation time; SQ: Semiquantitative.

(1): Dako, Glostrup, Denmark; (2): Leica Biosystems, Newcastle Upon Tyne, UK.

*: Proliferation Index: Low if < 14% and High if ≥14% positive nuclei were observed; #: score 3+ in cases with > 30% positive tumor cells; **: positive if at least moderate staining intensity in > 5% of tumor cells.

### Statistical analysis

Categorical data are presented as numbers and corresponding percentages, while continuous data are presented as median and range values. The Fisher's exact test or Pearson chi-square were used for group comparison of categorical data, while for continuous data the Mann-Whitney test was used. Distributional cut-offs were used to categorize tumors into low and high HER2 and TOP2A mRNA expression.

DFS was defined as the time interval from study entry to first locoregional recurrence, first distant metastasis, contralateral breast cancer, secondary neoplasm, death from the disease, or death from any cause non-related to breast cancer, whichever occurred first [30]. OS was measured from study entry until death from any cause. Surviving patients were censored at the date of last contact.

Kaplan-Meier curves and log-rank tests were used for comparing time to event distributions. Cox proportional hazard regression analyses, adjusted for treatment, were performed for the examined markers, as well as for the combination of HER2/TOP2A variables (HER2/TOP2A gene amplification, high HER2/TOP2A mRNA expression, HER2/TopoIIa protein positivity) to assess prognostic significance on OS and DFS. In multivariate analysis, a backward selection procedure with p > 0.10 as a removal criterion based on the likelihood ratio test was performed to identify significant clinicopathological variables among the following: age, treatment group (E-CMF vs. E-T-CMF), menopausal status (postmenopausal vs. premenopausal), tumor grade (III-undifferentiated vs. I-II), Ki67 protein expression (high vs. low), tumor size (> 5 cm vs. 2 to 5 cm vs. < 2 cm), number of positive axillary nodes (≥ 4 vs. 0-3), ER/PgR status (positive vs. negative), adjuvant hormonotherapy (yes vs. no), adjuvant radiotherapy (yes vs. no), type of operation (breast conserving surgery vs. modified radical mastectomy) and time interval from breast surgery operation (> 4 weeks vs. 2-4 weeks vs. < 2 weeks). Treatment group and the examined markers (or the combination of HER2/TOP2A) were entered in the final model, in order to examine whether they added independent prognostic information to the model containing the significant clinicopathological parameters.

The results of this study are presented according to reporting recommendations for tumor marker prognostic studies [31]. This study is prospective-retrospective as previously described [32]. The SPSS software was used for statistical analysis (SPSS for Windows, version 15.0, SPSS Inc.).

## Results

### Patient and Tumor Characteristics

In total 352 patients were included in the present analysis. Slightly more patients received adjuvant E-CMF chemotherapy (N = 193) than with E-T-CMF (N = 159). The majority of cases underwent modified radical mastectomy (78%) and had four or more involved nodes (75%). Most of the carcinomas were hormone-receptor positive (79%) and they also expressed high Ki67 (82%). With the exception of high tumor grade and HER2 protein overexpression, which were more common in the E-T-CMF arm, basic clinicopathological characteristics were well balanced when the 352 analyzed patients were stratified by adjuvant chemotherapy arm (Table 2). The unbalance of tumor grade in the two treatment arms had been reported in the randomized study [17]. After a median follow-up time of 98 months (range: 7.0-126.3), 125 patients (36%) had developed a documented disease relapse and 93 patients (26%) died. 5-year DFS and OS (E-CMF vs. E-T-CMF) was 68.7% vs. 72.1% (Hazard ratio [HR] = 1.12, 95% Confidence interval [CI]: 0.76-1.66, Wald's p = 0.56) and 5-year OS was 81.2% vs. 85.5% (HR = 1.31, 95% CI: 0.78-2.22, Wald's p = 0.31).

**Table 2:** Selective patient and tumor characteristics

	E-T-CMF		E-CMF		Analyzed cohort	
	(N = 159)		(N = 193)		(N = 352)	
	Median	Range	Median	Range	Median	Range
**Age **(in years)	50.2	23.8-75.9	50.6	22.5-78.0	50.6	22.5-78.0
**Number of nodes removed**	19	5-59	19	4-53	19	4-59
**Number of positive nodes**	7	0-54	6	0-49	6	0-54

	**N**	**%**	**N**	**%**	**N**	**%**

**Nodal involvement (n = 352)**						
0-3 nodes	34	21.4	54	28.0	88	25.0
≥ 4 nodes	125	78.6	139	72.0	264	75.0
**Menopausal status (n = 352)**						
Premenopausal	83	52.2	104	53.9	187	53.1
Postmenopausal	76	47.8	89	46.1	165	46.9
**Type of operation (n = 352)**						
Modified radical mastectomy	122	76.7	151	78.2	273	77.6
Breast conserving surgery	37	23.3	42	21.8	79	22.4
**Interval from operation (n = 352)**						
< 2 weeks	19	11.9	26	13.5	45	12.8
2-4 weeks	81	50.9	84	43.5	165	46.9
> 4 weeks	59	37.1	83	43.0	142	40.3
Tumor grade* (n = 351)						
I-II	64	40.5	112	58.0	176	50.1
III-Undifferentiated	94	59.5	81	42.0	175	49.9
**Tumor size (n = 352)**						
< 2 cm	45	28.3	67	34.7	112	31.8
2-5 cm	88	55.3	91	47.2	179	50.9
> 5 cm	26	16.4	35	18.1	61	17.3
**Adjuvant RT (n = 350)**						
No	27	17.1	39	20.3	66	18.9
Yes	131	82.9	153	79.7	284	81.1
**Adjuvant HT (n = 351)**						
No	12	7.6	21	10.9	33	9.4
Yes	146	92.4	172	89.1	318	90.6
**ER protein status (n = 306)**						
Negative	40	28.2	41	25.0	81	26.5
Positive	102	71.8	123	75.0	225	73.5
**PgR protein status (n = 305)**						
Negative	52	36.6	54	33.1	106	34.8
Positive	90	63.4	109	66.9	199	65.2
**Hormone receptor status (n = 307)**						
Negative	36	25.4	29	17.6	65	21.2
Positive	106	74.6	136	82.4	242	78.8
**Ki67 (n = 309)**						
Low (< 14%)	25	17.4	30	18.2	55	17.8
High (≥ 14%)	119	82.6	135	81.8	254	82.2
**HER2 CISH amplification (n = 285)**						
Non-amplified	100	75.2	121	79.6	221	77.5
Amplified	33	24.8	31	20.4	64	22.5
**HER2 mRNA expression (n = 314)**						
Low (< 75^th ^percentile)	108	75.0	128	75.3	236	75.2
High (≥ 75^th ^percentile)	36	25.0	42	24.7	78	24.8
HER2 protein expression (n = 297)**						
0-1+	86	63.2	125	77.6	211	71.0
2+	19	14.0	15	9.3	34	11.4
3+	31	22.8	21	13.0	52	17.5
**TOP2A CISH amplification (n = 266)**						
Non-amplified	110	90.9	137	94.5	247	92.9
Amplified	11	9.1	8	5.5	19	7.1
**TOP2A mRNA expression (n = 314)**						
Low (< median)	72	50.0	85	50.0	157	50.0
High (≥ median)	72	50.0	85	50.0	157	50.0
**TopoIIa protein status (n = 273)**						
Negative	43	35.2	56	37.1	99	36.3
Positive	79	64.8	95	62.9	174	63.7

*: p = 0.001; **: p = 0.023; The distribution of grade and HER2 protein expression differs significantly between the two groups.

RT. radiotherapy; HT. hormonotherapy; CISH. chromogenic in situ hybridization.

### HER2 and TOP2A gene status, mRNA and protein expression

**Figure 2:**
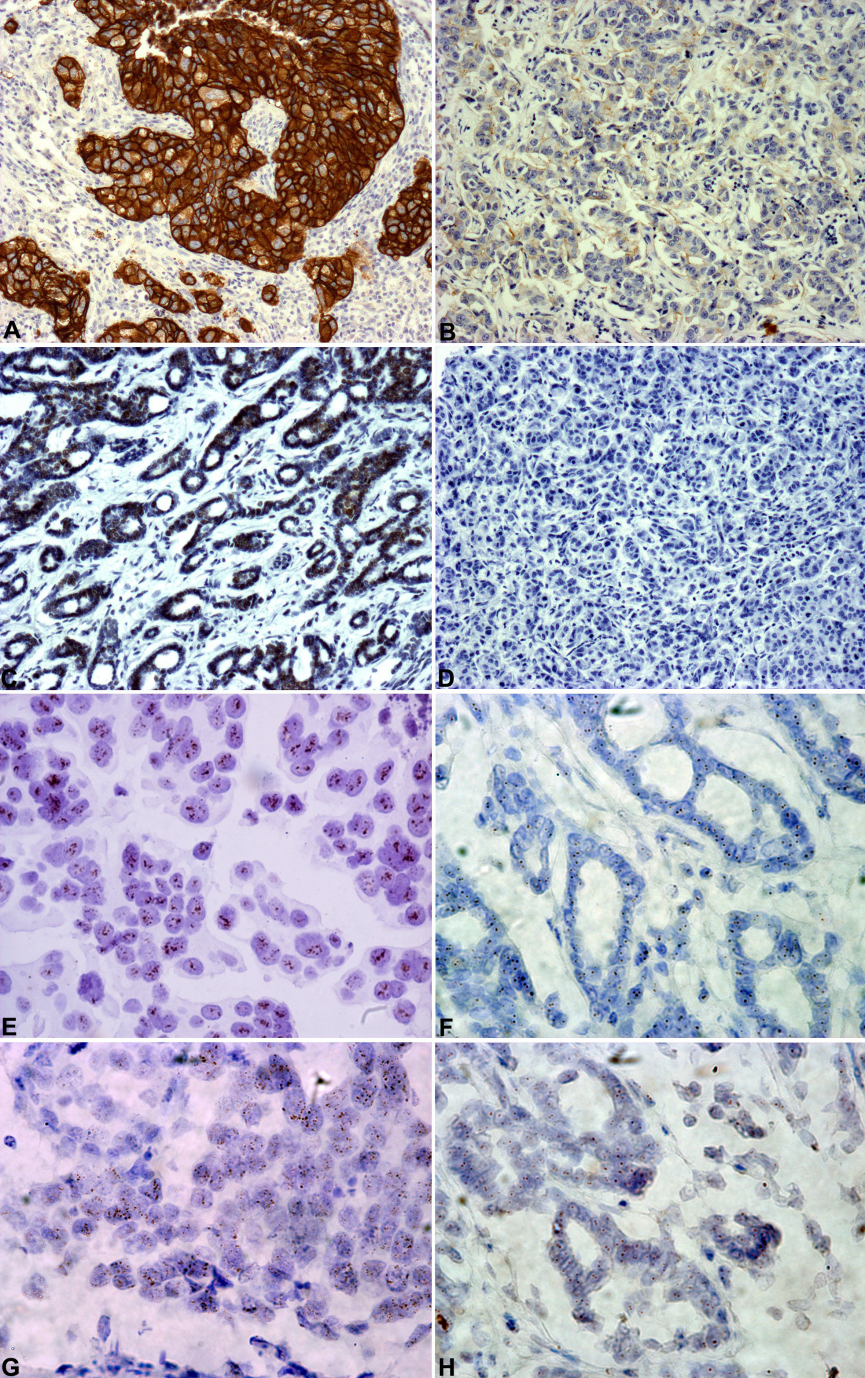
**Representative examples of HER2 and TopoIIa protein expression and corresponding gene status**. A-D: immunohistochemistry at a magnification of 100Χ (A: HER2 3+, B: HER2 0, C: TopoIIa positive and D: TopoIIa negative). E-H: CISH at a magnification of 600Χ (E: HER2 positive/amplified, F: HER2 negative/diploid, G: TOP2A positive and H: TOP2A negative).

**Figure 3:**
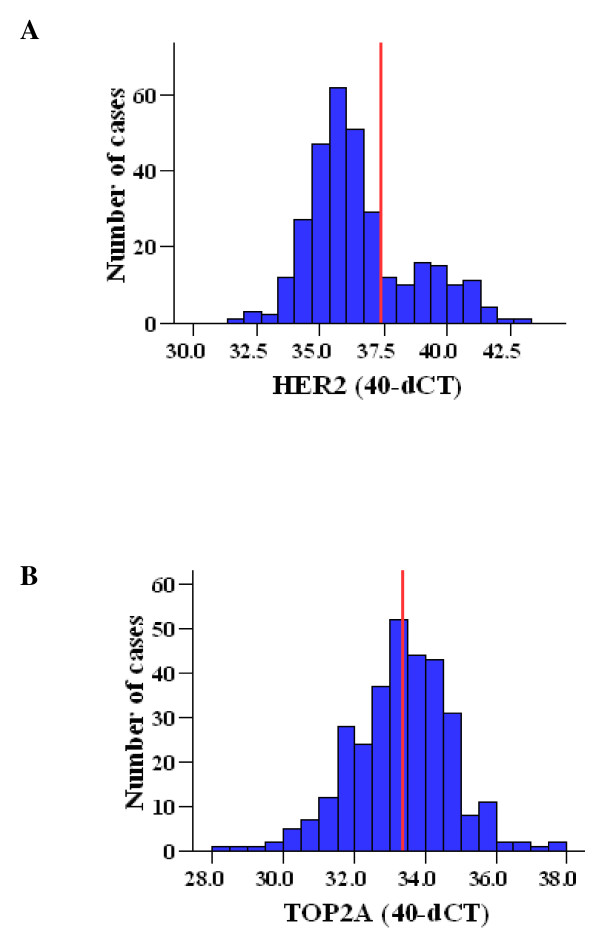
**Distribution of relative mRNA expression (40-dCT values) for A) HER2 (median: 36.02, 25th percentile: 35.20, 75th percentile: 37.41) and B) TOP2A (median: 33.38, 25th percentile: 32.46, 75th percentile: 34.18)**. Red lines represent the cut-offs used (75th percentile for HER2 and median for TOP2A).

CISH analysis for HER2 was informative in 285 cases (Figure 1, Table 2) with representative examples shown in Figure 2A and 2B. HER2 gene amplification (low and high) was observed in 64 cases (22.5%). Polysomy with 3 to 5 signals was observed in 60 cases, while the rest 161 cases were diploid. Relative quantification (RQ) of HER2 mRNA expression was informative in 314 cases. The distribution of HER2 RQ values was bimodal (Figure 3A) and thus the 75^th ^percentile (37.41 arbitrary units) was used to classify HER2-low and HER2-high expressing tumors, since it was close to the natural cut-off. IHC analysis for HER2 was applicable in 297 cases (Figure 1, 2E and 2F) with the majority of cases (71%) scored as HER2 0-1+, 17.5% as 3+, and 11% as 2+ (Table 2).

**Figure 4:**
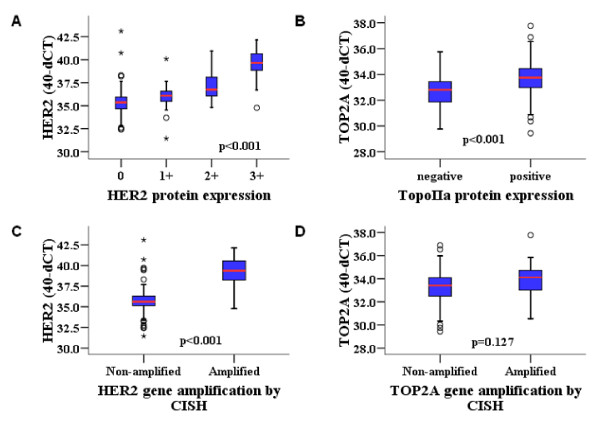
**Association of HER2 and TOP2A relative mRNA expression (40-dCT values) with corresponding protein expression by IHC (A, B) and gene amplification by CISH (C, D)**. Comparisons were made using Kruskal Wallis and Mann-Whitney tests.

HER2 gene amplification was significantly associated with HER2 immunostaining (p < 0.001), with only two HER2 non-amplified tumors showing 3+ staining for the corresponding protein, which proves the strong association between HER2 gene amplification and protein overexpression. Increased HER2 mRNA expression was found significantly more frequent in IHC grade 3 tumors (Figure 4A) and was also associated with HER2 gene amplification Figure 4B). The same significance was obtained for binary high HER2 mRNA with IHC and with HER2 gene status (Fisher's exact test p < 0.001).

CISH, mRNA RQ values and IHC results for TOP2A were informative in 266, 314 and 273 cases, respectively (Figure 1, Table 2). Representative examples for TOP2A gene status with CISH are shown in Figure 2C and 2D. TOP2A gene amplification was observed in 19 cases (7%) (5 cases with high and 14 cases with low gene amplification). TOP2A polysomy was seen in 51 cases (19%). No deletions were identified. In contrast to HER2, TOP2A mRNA expression had a unimodal distribution (Figure 3B) and thus the median cut-off (33.38 arbitrary units) was used to classify low and high TOP2A mRNA expression. Positive TopoIIa protein immunostaining was observed in 64% of the cases (Table 2). Representative examples of TopoIIa IHC are shown in Figure 2G and 2H.

TOP2A gene amplification was not associated with TopoIIa protein status (p = 0.80), since the majority (92%) of TopoIIa protein positive tumors were TOP2A non-amplified and IHC positivity rates did not differ in amplified and non-amplified tumors. Increased TOP2A mRNA expression, both as a continuous (Figure 4C) and as a binary variable, was strongly associated with positive TopoIIa IHC status (Fisher's exact test, p < 0.001 for the binary comparison). By contrast, TOP2A mRNA expression was not associated with TOP2A gene amplification (Figure 4D and Fisher's exact test p = 0.395 for the binary comparison). Only one TOP2A amplified tumor exhibited a higher mRNA RQ value in comparison to non-amplified tumors.

### Association between HER2 and TOP2A parameters

TOP2A gene status was strongly associated with HER2 gene status (Fisher's exact test, p < 0.001). Co-amplification of HER2 and TOP2A genes was observed in 13 out of 282 informative tumors (4.6%) and in 13/61 HER2 positive tumors informative for TOP2A (21.3%). TOP2A amplification without HER2 amplification was observed in 6 cases (31.6% of all TOP2A amplified cases, 2.3% among all informative tumors for both HER2 and TOP2A). Upon revisiting these six cases, four displayed very low HER2 gene amplification (3 to 5 copies per nucleus), while 3 of them were also centromere 17 polysomic. According to current guidelines for assessing HER2 amplification with CISH [23], these cases had been correctly characterized as HER2 non-amplified; however, they did contain an increased number of HER2 copies. Hence, only two cases in our series remained as unequivocally low amplified for TOP2A with diploid HER2 and centromere 17 (0.8% among all informative tumors or 10% among TOP2A amplified tumors). Such rates have previously been reported for TOP2A amplification in the absence of HER2 amplification, when tested with FISH [33].

When compared in a continuous scale, mRNA expression of both genes exhibited a significant, although linearly weak correlation (Rho = 0.23, p < 0.001). However, when examined as binary variables (high/low mRNA expression), HER2 and TOP2A RNA expression were not associated (Fisher's exact test, p = 0.36). Among the 314 cases with informative mRNA results for both HER2 and TOP2A, 43 cases (13.7%) expressed both genes at high levels.

Among the HER2 IHC positive cases, 66% were also TopoIIa protein positive, while among all tumors informative for IHC, 33 cases were HER2 and TopoIIa positive (9.4%). Hence, the overall association of TopoIIa with HER2 protein positivity was not significant (Fisher's exact test, p = 0.75).

**Figure 5:**
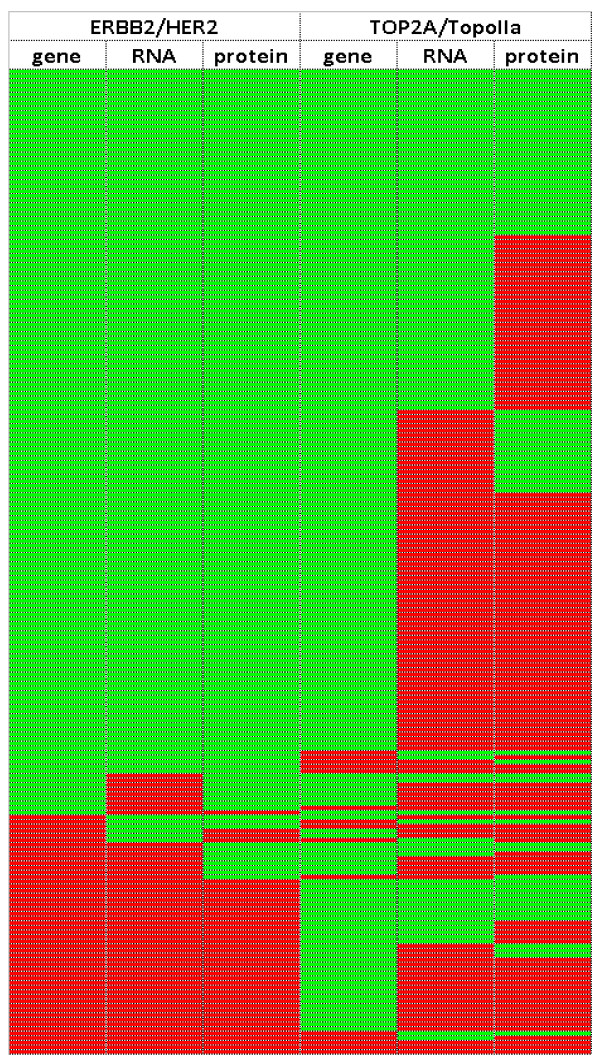
**HER2 and TOP2A profiling for gene status, RNA and protein expression in adjuvantly treated breast carcinomas**. Green cells: non-amplified, low mRNA expression, negative protein expression; Red cells: gene amplification, high mRNA expression, protein positivity. HER2 mRNA and protein expression exhibit different positivity patterns in comparison to TOP2A mRNA and TopoIIa protein expression. The majority of tumors with high TOP2A mRNA expression (112/121 [95.6%]) and the majority of tumors positive for TopoIIa protein expression (145/158 [91.8%]) were not amplified.

HER2 and TOP2A profiles for the 214 tumors informative for all above parameters (61% of the entire tumor series) are shown in Figure 5.

### Association of HER2 and TOP2A with clinicopathological parameters

Patient age, menopausal status and lymph node involvement were not associated with HER2 and TOP2A parameters in the cohort examined. High-grade tumors expressed higher levels of HER2 and TOP2A mRNA (p = 0.001 and p = 0.002, respectively) in comparison to lower grade tumors. In addition, although HER2 3+ tumors were more often of high grade, this feature did not reach statistical significance (p = 0.066), but significantly more high-grade tumors were positive for TopoIIa protein (p < 0.001). TOP2A gene amplification occurred independently of tumor grade. Further, TOP2A gene amplification was preferably observed in tumors > 2 cm (16/19 amplified cases [84%], p = 0.085); similarly, HER2 protein positivity occurred more often in tumors > 2 cm (p = 0.078).

Associations of HER2 and TOP2A parameters with ER and PgR status, as well as with Ki67 scores are presented in Table 3. HER2 3+ tumors significantly more often exhibited negative ER status, negative PgR status, and Ki67 score ≥ 14%. Similar results were obtained for HER2 amplification with CISH in comparison to the same markers, as well as for HER2 mRNA expression regarding ER and PgR status. Regarding TOP2A, TopoIIa protein positivity and high TOP2A mRNA expression were significantly associated with high Ki67 labeling. No association was found for these parameters, as well as for TOP2A gene amplification with ER and PgR tumor status.

**Table 3:** Associations of HER2 and TOP2A with ER, PgR and Ki67 status (assessed by IHC)

	ER status			PgR status			Ki67 status		
	Negative	Positive	p	Negative	Positive	p	Low	High	p
	N (%)	N (%)		N (%)	N (%)		N (%)	N (%)	
**HER2 (CISH)**			< 0.001			0.001			0.035
Non-amplified	47 (61.8)	173 (83.2)		63 (65.6)	156 (83.4)		42 (89.4)	176 (75.2)	
Amplified	29 (38.2)	35 (16.8)		33 (34.4)	31 (16.6)		5 (10.6)	58 (24.8)	
**HER2 (mRNA)**			< 0.001			< 0.001			0.086
Low (< 75^th ^perc)	38 (55.9)	166 (82.6)		53 (59.6)	151 (84.4)		40 (87.0)	168 (74.0)	
High (≥ 75^th ^perc)	30 (44.1)	35 (17.4)		36 (40.4)	28 (15.6)		6 (13.0)	59 (26.0)	
**HER2 (IHC)**			0.003			0.002			0.016
0-1+	47 (58.8)	163 (75.5)		61 (58.7)	148 (77.5)		43 (87.8)	163 (67.4)	
2+	9 (11.3)	25 (11.6)		15 (14.4)	19 (9.9)		3 (6.1)	31 (12.8)	
3+	24 (30.0)	28 (13.0)		28 (26.9)	24 (12.6)		3 (6.1)	48 (19.8)	
**TOP2A (CISH)**			0.29			0.46			0.33
Non-amplified	64 (90.1)	182 (93.8)		83 (91.2)	162 (93.6)		41 (97.6)	202 (91.8)	
Amplified	7 (9.9)	12 (6.2)		8 (8.6)	11 (6.4)		1 (2.4)	18 (8.2)	
**TOP2A (mRNA)**			0.99			0.20			0.003
Low (< median)	34 (50.0)	100 (49.8)		39 (43.8)	94 (52.5)		33 (71.7)	107 (47.1)	
High (≥ median)	34 (50.0)	101 (50.2)		50 (56.2)	85 (47.5)		13 (28.3)	120 (52.9)	
**TopoIIa (IHC)**			0.39			0.99			< 0.001
Negative	29 (40.3)	69 (34.5)		33 (35.9)	64 (35.8)		32 (78.0)	66 (28.8)	
Positive	43 (59.7)	131 (65.5)		59 (64.1)	115 (64.2)		9 (22.0)	163 (71.2)	

### Prognostic/predictive significance of HER2 and TOP2A

**Figure 6:**
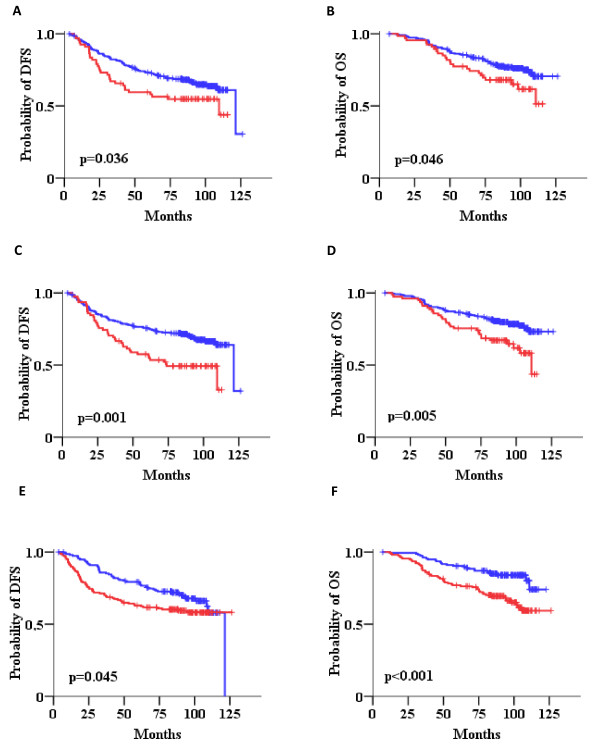
**Impact of HER2 gene amplification and HER2 and TOP2A mRNA expression on patient outcome**. Kaplan-Meier curves for HER2 status (A, B), HER2 mRNA expression (C, D) and TOP2A mRNA expression (E, F) in terms of disease-free (DFS) and overall survival (OS). Comparisons by log-rank test. Red lines: positive or high; Blue lines: negative or low.

Univariate Cox regression analysis (Table 4), adjusted for paclitaxel treatment, revealed increased risk of relapse for HER2 IHC grade 3+ in comparison to 0 or 1+ tumors, as well as for tumors with HER2 gene amplification in comparison to non-amplified tumors. Since we performed IHC and CISH analysis, we also examined the prognostic significance of the combination for the two methods. Thus, we introduced a HER2 status variable considering IHC 3+ and/or CISH amplified tumors as HER2 positive; it should be noted however, that for 12 of the 297 patients with HER2 IHC data, CISH data were not available. The remaining patients were considered as HER2 negative. Again, positive HER2 status was associated with increased risk for relapse in comparison to negative HER2 status (Table 4, Figure 6A). In addition, high HER2 mRNA expression was strongly associated with shorter DFS (Table 4, Figure 6C). Among TOP2A parameters, increased risk for relapse was only observed for tumors with high TOP2A mRNA expression as compared to tumors expressing low TOP2A (Figure 6E). In terms of OS, HER2 IHC 3+ score was associated with increased risk for death, while IHC 2+ tumors also performed worse as compared to 0 or 1+ tumors. HER2 gene status by CISH was marginally not associated with OS, while HER2 IHC positive tumors and tumors expressing high HER2 mRNA expression were also associated with increased risk for death (Figure 6B and 6D). High TOP2A mRNA expression was strongly adversely associated with survival (Table 4, Figure 6F). In order to examine the predictive significance of HER2 and TOP2A expression we tested the interaction with paclitaxel treatment but no such association was found (Wald's p > 0.05 in all tests).

**Table 4:** Univariate Cox regression analysis for HER2 and TOP2A adjusted for treatment

	Disease-free survival			Overall survival		
	
	HR	**95% C.I**.	Wald's p	HR	**95% C.I**.	Wald's p
**HER2 (CISH)**						
Non-amplified	1			1		
Amplified	1.55	1.01-2.39	**0.045**	1.62	0.98-2.66	0.060
**HER2 (mRNA)**						
Low	1			1		
High	1.89	1.29-2.79	**0.001**	1.91	1.21-3.02	**0.005**
**HER2 (IHC)**						
0-1+	1			1		
2+	1.61	0.93-2.78	0.091	2.13	1.18-3.84	**0.012**
3+	1.75	1.10-2.79	**0.019**	1.78	1.02-3.08	**0.041**
**HER2 status**						
Negative	1			1		
Positive*	1.57	1.04-2.39	**0.033**	1.68	1.04-2.72	**0.034**

**TOP2A (CISH)**						
Non-amplified	1			1		
Amplified	0.35	0.11-1.10	0.072	0.34	0.08-1.38	0.131
**TOP2A (mRNA)**						
Low	1			1		
High	1.45	1.01-2.10	**0.046**	2.34	1.46-3.73	**< 0.001**
**TopoIIa (IHC)**						
Negative	1			1		
Positive	1.37	0.90-2.10	0.145	1.55	0.93-2.60	0.094

**HER2/TOP2A CISH**						
All other	1			1		
Both amplified	0.17	0.2-1.20	0.075	0.25	0.04-1.80	0.17
**HER2/TOP2A mRNA**						
All other	1			1		
Both high	2.15	1.37-3.38	**0.001**	2.51	1.51-4.18	**< 0.001**
**HER2/TopoIIa IHC**						
All other	1			1		
Both positive	1.92	1.14-3.25	**0.015**	1.89	1.04-3.44	**0.038**

* IHC 3+ or CISH amplified.

There was no interaction between examined markers with treatment regimens (p > 0.05 for all interaction tests).

**Figure 7:**
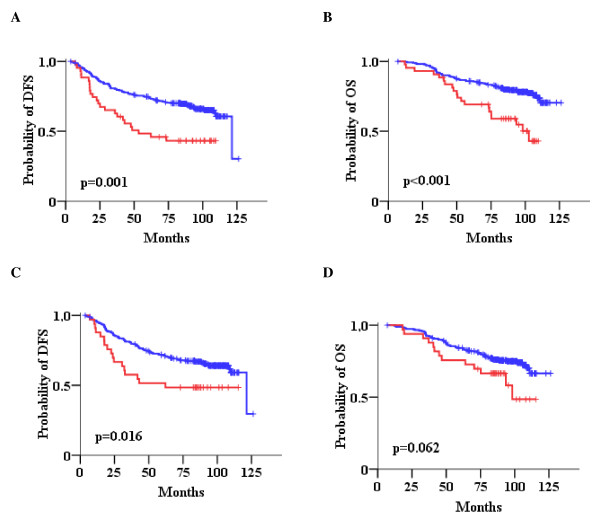
**Impact of combined HER2 and TOP2A (HER2/TOP2A) parameters on patient outcome**. Kaplan-Meier curves for combined HER2/TOP2A mRNA expression (A, B) and combined HER2/TopoIIa protein status (C, D) in terms of DFS and OS. Comparisons by log-rank test. Red lines: HER2/TOP2A high mRNA expression (A, B) or HER2/TopoIIa positive protein expression (C, D); Blue lines: all other combinations.

We next analyzed combined qualitative positivity of HER2/TOP2A variables at the same molecular level (HER2/TOP2A gene amplification, HER2/TOP2A high mRNA as a binary variable, HER2 3+/TopoIIa protein positivity). This approach revealed that patients with tumors expressing both HER2/TOP2A high mRNA, and to a lesser extent, positive for both HER2/TopoIIa proteins had a significantly worse outcome than patients with all other combinations of these two parameters (both low or low/high for mRNA; both negative or negative/positive for protein) (Table 4). Forty-three patients with high HER2/TOP2A mRNA expression had a higher risk for relapse and death in comparison to patients with tumors expressing all other combinations of HER2/TOP2A mRNA expression (Figure 7A and 7B). Patients with tumors positive for both HER2 and TopoIIa proteins (n = 33) were in higher risk for relapse and death in comparison to patients with tumors exhibiting all other patterns of HER2/TopoIIa IHC status (Figure 7C and 7D). Finally, patients with tumors carrying HER2/TOP2A co-amplification showed a trend for favorable disease-free survival in comparison to tumors where only one or none of the two genes was amplified. It must be noticed that HER2/TOP2A co-amplified tumors in this study concerned 13 patients only, of which only one relapsed at 18 months and died of disease at 48 months, while no other events were noted during the entire follow-up period (101.2 months).

In the multivariate analysis we first examined the prognostic significance of single HER2 and TOP2A parameters adjusted for significant clinicopathological variables (Table 5). Among HER2 variables, only HER2 mRNA expression remained significant for both DFS and OS, with tumors expressing high mRNA levels having increased risk for relapse and death, as compared to low HER2 mRNA tumors. Among the three parameters examined for TOP2A, gene amplification was associated with longer DFS, while tumors with high TOP2A mRNA expression had shorter survival. These findings may appear as discrepant but in fact they are not, because most of the tumors with high TOP2A mRNA expression were not TOP2A amplified (Figure 5), while only 9/19 TOP2A amplified tumors expressed high TOP2A RNA. Thus, TOP2A amplified and high TOP2A mRNA tumors correspond to distinct groups of patients with minimal overlap, especially with respect to the second category. In the final model we included the significant predictors for DFS and OS (mRNA expression for HER2 and TOP2A, and CISH gene status for TOP2A) and found that only HER2 mRNA expression was a significant adverse prognostic factor for both DFS and OS, while TOP2A mRNA expression was significant only for OS. In this model, nodal involvement and hormonal status, as well as Ki67 labeling remained significant in predicting patient DFS and OS.

**Table 5:** Multivariate Cox regression analysis A) for HER2, B) for TOP2A and C) for both HER2 and TOP2A, adjusted for significant clinicopathological parameters in terms of DFS and OS

		Disease-free survival			Overall survival		
		HR	**95% C.I**.	Wald's p	HR	**95% C.I**.	Wald's p
**A)**	**Group**						
	E-CMF vs E-T-CMF	1.07	0.72-1.59	0.75	1.58	0.96-2.60	0.071
	**Tumor size**						
	2-5 cm vs < 2 cm	1.62	0.99-2.67	0.056			
	> 5 cm vs < 2 cm	1.73	0.92-3.22	0.087			
	**Adjuvant HT**						
	Yes vs No	0.50	0.27-0.94	**0.031**			
	**Nodal involvement**						
	≥ 4 vs 0-3	2.16	1.19-3.93	**0.011**	1.83	0.92-3.61	0.082
	**Hormone receptor status**						
	Positive vs Negative				0.54	0.30-0.94	**0.030**
	**Ki67**						
	High vs Low	1.69	0.91-3.15	0.096	3.54	1.27-9.84	**0.015**
	**HER2 (mRNA)**						
	High vs Low	1.68	1.10-2.58	**0.016**	1.77	1.04-3.02	**0.035**

**B)**	**Group**						
	E-CMF vs E-T-CMF	1.20	0.79-1.82	0.40	1.58	0.96-2.60	0.071
	**Nodal involvement**						
	≥ 4 vs 0-3	3.03	1.56-5.85	**0.001**	2.21	1.11-4.38	**0.024**
	**Hormone receptor status**						
	Positive vs Negative	0.49	0.31-0.78	**0.003**	0.42	0.24-0.71	**0.001**
	**Ki67**						
	High vs Low	2.04	1.05-3.97	**0.035**	3.50	1.25-9.76	**0.017**
	**TOP2A (CISH)**						
	Amplified vs Non-amplified	0.29	0.09-0.93	**0.038**			
	**TOP2A (mRNA)**						
	High vs Low				2.08	1.24-3.47	**0.005**

**C)**	**Group**						
	E-CMF vs E-T-CMF	1.17	0.74-1.84	0.51	1.60	0.97-2.64	0.065
	**Nodal involvement**						
	≥ 4 vs 0-3	2.82	1.39-5.71	**0.004**	2.09	1.05-4.16	**0.036**
	**Hormone receptor status**						
	Positive vs Negative	0.56	0.33-0.96	**0.035**	0.49	0.28-0.86	**0.014**
	**Ki67**						
	High vs Low	2.24	1.01-4.98	**0.048**	3.18	1.13-8.92	**0.028**
	**HER2 (mRNA)**						
	High vs Low	1.80	1.10-2.93	**0.019**	1.73	1.02-2.93	**0.040**
	**TOP2A (CISH)**						
	Amplified vs Non-amplified	0.36	0.11-1.17	0.091			
	**TOP2A (mRNA)**						
	High vs Low	1.52	0.97-2.41	0.070	2.06	1.23-3.46	**0.006**

HT, hormonotherapy

The same approach of multivariate analysis was applied for the combined HER2/TOP2A variables (Table 6). As shown, HER2/TOP2A co-amplification and HER2/TopoIIa protein co-expression were significant for DFS but not for OS, while high HER2/TOP2A mRNA co-expression was strongly adversely significant for both DFS and OS. When the analysis was adjusted for all combined parameters along with classic clinicopathological variables (nodal involvement, hormone receptor status and Ki67 labeling), high HER2/TOP2A mRNA co-expression remained strongly significant as an unfavorable prognostic factor for both DFS and OS, while HER2/TOP2A gene co-amplification remained significant as a favorable prognostic factor for OS only. Again, throughout the applied models, hormone receptor status was a favorable, while the number of involved lymph nodes and high Ki67 protein expression were unfavorable independent predictors of patient outcome.

**Table 6:** Multivariate Cox regression analysis A) for HER2/TOP2A co-amplification, B) for HER2/TOP2A mRNA expression, C) for HER2/TopoIIa protein expression and D) for all combined HER2/TOP2A parameters, adjusted for significant clinicopathological parameters in terms of DFS and OS

		Disease-free survival			Overall survival		
		
		HR	**95% C.I**.	Wald's p	HR	**95% C.I**.	Wald's p
**A)**	E-CMF vs E-T-CMF	1.26	0.85-1.88	0.25	1.67	1.03-1.72	**0.038**
	Nodal involvement (≥ 4 vs 0-3)	3.53	1.82-6.85	**< 0.001**	2.96	2.96	**0.004**
	Hormone receptor status (pos. vs neg.)	0.48	0.31-0.77	**0.002**	0.40	0.24-0.68	**0.018**
	Ki-67 (high vs low)	1.97	1.06-3.65	**0.031**	2.77	1.19-6.46	**0.018**
	HER2&TOP2A gene (both ampl. vs all other)	0.12	0.02-0.88	**0.037**	0.16	0.02-1.14	0.067

**B)**	E-CMF vs E-T-CMF	1.16	0.77-1.75	0.47	1.56	0.95-2.57	0.079
	Nodal involvement (≥ 4 vs 0-3)	2.67	1.44-4.93	**0.002**	2.07	1.05-4.09	**0.035**
	Hormone receptor status (pos. vs neg.)	0.57	0.36-0.91	**0.018**	0.48	0.28-0.83	**0.008**
	Ki67 (high vs low)	1.92	0.98-3.77	0.057	3.40	1.22-9.48	**0.019**
	HER2&TOP2A mRNA (high vs low)	2.30	1.39-3.81	**0.001**	2.48	1.41-4.36	**0.002**

**C)**	E-CMF vs E-T-CMF	1.18	0.80-1.74	0.39	1.62	1.02-2.57	**0.042**
	Nodal involvement (≥ 4 vs 0-3)	3.00	1.64-5.49	**< 0.001**	2.43	1.25-4.73	**0.009**
	Hormone receptor status (pos. vs neg.)	0.64	0.41-0.99	**0.047**	0.51	0.31-0.85	**0.009**
	Ki67 (high vs low)	1.68	0.93-3.05	0.086	2.27	1.03-5.00	**0.041**
	HER2&TopoIIa IHC (both pos. vs all other)	1.77	1.04-3.05	**0.034**	1.58	0.86-2.91	0.14

**D)**	E-CMF vs E-T-CMF	1.13	0.73-1.75	0.59	1.53	0.89-2.26	0.12
	Nodal involvement (≥ 4 vs 0-3)	3.62	1.78-7.34	**< 0.001**	2.87	1.29-6.40	**0.010**
	Hormone receptor status (pos. vs neg.)	0.51	0.31-0.84	**0.009**	0.45	0.25-0.81	**0.008**
	Ki67 (high vs low)	2.05	0.99-4.21	0.051	4.35	1.34-14.18	**0.015**
	HER2&TOP2A gene (both ampl. vs all other)	0.13	0.02-0.96	**0.046**	0.23	0.03-1.74	0.15
	HER2&TOP2A mRNA (high vs low)	2.41	1.31-4.42	**0.005**	2.83	1.42-5.63	**0.003**
	HER2&TopoIIa IHC (both pos. vs all other)	1.36	0.68-2.70	0.38	0.92	0.42-2.18	0.92

## Discussion

Even though the adverse prognostic role of HER2 parameters (gene amplification and mRNA and protein overexpression) in patients with operable breast cancer is indisputable, that of TOP2A parameters remains controversial. Moreover, the optimal parameter(s) and respective methodologies for assessing HER2 [23,34,35] and TOP2A [7,36] status are still not well defined. Concerning methods, in this study we employed CISH for the assessment of HER2 and TOP2A gene status. CISH is an accepted method for the evaluation of the HER2 gene [23] but it should be noticed that, while CISH and FISH results are highly concordant in true negative (diploid) and highly amplified tumors, the two methods yield discordant results in cases with low HER2 amplification [37]. Further, CISH is not the method for reporting gene deletions [16,38], hence we did not register any TOP2A deletions like those reported with FISH [33,39-41]. In terms of TOP2A gene amplification, the incidence with CISH in our cohort (7%) was low but still comparable to previous reports assessing this parameter with FISH [33,42],. while TOP2A amplified/HER2 non-amplified tumors have also been reported with FISH [33]. It seems that, concerning the amplification part of TOP2A gene pathology, CISH results may be considered comparable with the much more abundant FISH results in the literature.

It is noteworthy that information in the literature regarding a thorough evaluation of both HER2 and TOP2A at all levels of molecular pathology (gene, RNA, protein) is very limited. To our knowledge the present study is one of the few attempting to define the relationship between TOP2A gene copy numbers with TOP2A RNA and TopoIIa protein expression and possibly the first in the context of an adjuvant phase III trial including patients with breast cancer treated with anthracyclines. In a report by Brase et al [36], the prognostic relevance of the HER2 and TOP2A genes was comprehensively studied at the DNA, RNA and protein level in three independent patient cohorts. It is of interest, that although this report differs from ours substantially in terms of design, patient population and treatment, both studies share a considerable number of common findings. In the Brase et al study [36], high TOP2A RNA levels were significantly associated with shorter metastasis-free survival in node-negative (low-risk) patients who did not receive adjuvant chemotherapy, but the same parameter seemed to be associated with better response to anthracyclines. In the present study, all patients had received epirubicin; in this "anthracycline-treatment homogeneous" population, where the parameter "anthracyclines" may be eliminated, high TOP2A mRNA expression was an adverse prognostic parameter for patient OS, a function that remained significant after adjustment for hormone receptor status, nodal involvement, Ki67 labelling and HER2 status. Thus, the present data support an unfavorable long-term prognostic role for TOP2A mRNA expression in high-risk patients who received adjuvant treatment.

In contrast to TOP2A mRNA expression, TOP2A gene amplification was independently associated with prolonged DFS in our patients, in line with repeated reports pointing out TOP2A gene amplification as a marker predicting for response to anthracyclines [42-47]. Of note, since, as shown, tumors with TOP2A gene amplification did not necessarily express high TOP2A mRNA levels and *vice versa*, the analyzed groups of patients with TOP2A gene amplification and high TOP2A mRNA expression overlapped to a small degree (8%) but certainly did not match. The effect of TOP2A amplification on patient outcome depends on the setting examined and seems relevant mainly in HER2-positive tumors, as revealed in the largest series tested so far for both HER2 and TOP2A gene amplification [42]. The incidence of TOP2A-amplified tumors within the HER2-positive group was relatively small in the present study (21%), instead of the ~35% reported by Press et al [42]. Even in this small group of patients, HER2/TOP2A co-amplification was revealed as an independent good prognostic factor for DFS in multivariate analysis; since DFS is treatment-related, this favorable prognostic value of HER2/TOP2A co-amplification might be considered in line with the earlier described positive predictive value of this marker for anthracyclines. Whether TOP2A itself or some other gene in the same chromosomal region, which is also detected by the large CISH/FISH probes, is responsible for the favourable prognostic value of HER2/TOP2A co-amplification remains to be elucidated.

As discussed above, two different TOP2A parameters, gene amplification and mRNA expression, seem to have a distinct impact on the outcome of breast cancer patients treated in the adjuvant setting. Although this appears as a paradox, it is fully compatible with the descriptive data presented in the present study concerning associations among TOP2A parameters: TOP2A mRNA and TopoIIa protein expression were strongly associated with each other but largely unrelated to TOP2A gene status. In comparison, all HER2 parameters (gene status, mRNA and protein expression) were strongly interrelated in our series, in accordance to most published data so far (comprehensively reviewed in [48]). The lack of associations between TOP2A gene status and TOP2A gene products (RNA and protein) has repeatedly been reported [27,36,43,46,49-51] and does not seem surprising considering key issues in TOP2A regulation and function. There is increased demand for TopoIIa protein during DNA replication [52], hence, the corresponding gene is transcribed independently of the existing copy numbers or of activated oncogenes [53]. In addition, TOP2A transcription may be strongly downregulated by wild type p53 [54], or strongly upregulated by HMGB1 and HMGB2 [55], while the half-life of TOP2A mRNA may depend on redox-sensitive protein complexes [56]. Biologically, the effective molecule is the protein and not its precursor nucleic acids but a prognostic role for TopoIIa protein could not be demonstrated so far in large patient series, probably because of differences in the performance and evaluation of immunohistochemical assessments (reviewed in [57]). Nevertheless, TOP2A mRNA levels were strongly associated with TopoIIa protein expression in our series, which at least indicates effective translation of TOP2A mRNA in the majority of the cases.

In line with the role of TOP2A in cell proliferation (reviewed in [12,58]), we observed strong associations of the proliferation marker Ki67 and tumor grade (which is characterized by Ki67 labelling) with TopoIIa protein and TOP2A mRNA levels. High Ki67 scores coinciding with TopoIIa protein (over)expression have been reported for breast carcinomas as reflecting tumor proliferation status [50,52,57,59], while the adverse prognostic impact of Ki67 in breast cancer is well established (reviewed in [60]). With respect to the strong association between Ki67 and TOP2A mRNA, which was also reported in a microarray profiling study [61], it might be argued that the herein observed adverse prognostic value of TOP2A mRNA reflects its strong association with high proliferation rates. This may in part be true, as previously suggested for TopoIIa protein as well [62]. However, while Ki67 was an unfavorable prognosticator for both DFS and OS, the adverse prognostic effect of TOP2A mRNA was limited to OS. In fact, this latter finding was the only contrasting point between the present study involving high-risk early breast cancer in comparison to low-risk early breast cancer, where TOP2A mRNA was unfavourably associated with metastasis-free survival [36]. To understand the long term influence of TOP2A expression on patient outcome, we need to consider newer functional aspects of TopoIIa protein and how these affect the fate of cancer cells when attacked by cytotoxic agents, such as anthracyclines (reviewed in [58,63]). It seems that, according to its molecular environment (among other parameters, TopoIIb and DNA repair capacity), TopoIIa protein may contribute to the large array of genomic aberrations observed in advanced cancers, including alterations of the TOP2A gene itself, as they are found at increasing rates with progressing tumor size ([64] and present study). At present, several questions regarding TOP2A in cancer cells remain unanswered, such as, how are TOP2A amplified genes regulated, whether genes are amplified in one piece or in fragments, or whether they lack regulatory regions, what are the genomic profiles of relapsed/metastatic vs. primary tumors, especially if treatment has been administered, and so on. Genomic profiles of higher resolution than obtained with FISH/CISH probes (which detect additional genes than the ones they are meant for anyway) would be required for these studies, while interesting data on genomic patterns in relevance to breast cancer subtypes have already been offered [65] and await further evaluation.

In terms of predicting response to paclitaxel, none of the TOP2A parameters examined was related to patient outcome. This was somehow expected, since the addition of this drug to the epirubicin containing treatment schemes did not offer a significant advantage for disease-free or overall survival in the same patient cohort [17], while no association was observed between TOP2A status and response to taxanes in the neo-adjuvant setting as well [66]. HER2 status (gene amplification and/or protein overexpression) and HER2 mRNA expression were not related to benefit from the taxane treatment in the present study. Relevant HER2 mRNA expression data could not be retrieved from the literature. The present HER2 status data seem to be in contrast to previous reports [8,66], as well as to the findings from the CALGB 9344/INT0148 adjuvant trial on node-positive patients who were treated with doxorubicin/cyclophosphamide with or without paclitaxel, probably due to clinical context, methodological and treatment administration differences. In addition, Ki67 was also not associated with response to paclitaxel in our series, which does not support a previously suggested taxane-predictive role of this marker [60].

The adverse prognostic impact of high HER2 mRNA expression, for the same patient cohort, has already been published by our group [67]. In comparison to HER2 mRNA, HER2 status was associated with patient outcome as a single variable but lost its prognostic significance upon multivariate analysis, in line with previous reports in the same treatment setting [39,68,69]. It seems that HER2 mRNA remains the most significant prognostic HER2 parameter, and various assays for the relative quantification of HER2 mRNA expression by qRT-PCR, in the single [26,48,70] or multiplex [71,72] mode have already been developed and evaluated, while HER2 mRNA is included in the Recurrence Score obtained by Oncotype DX [73]. In this study, high HER2 mRNA expression was an independent unfavorable prognostic factor, especially in terms of predicting relapse. Most interestingly, though, when examining high mRNA expression for both HER2 and TOP2A as a binary variable in the same tumor, this co-expression marker was strongly associated with shorter disease-free and overall survival, while it emerged as a new independent adverse prognostic factor in adjuvantly treated breast cancer patients. Of note, although the HER2 part of the high HER2/TOP2A mRNA co-expression marker is related to HER2 gene amplification, the TOP2A part is evidently not, as described throughout this manuscript. The biological background underlying this interaction is currently unknown and may be related to the conditions driving TOP2A (over)expression in the absence of gene amplification, as described above. It should also be noted that this combined marker is based on cohort-dependent cut-offs that were set for defining high mRNA expression for HER2 and TOP2A; hence, its validation in independent and larger patient cohorts is mandatory.

## Conclusion

In conclusion, this study confirms the favorable prognostic value of HER2/TOP2A co-amplification and the adverse prognostic value of high TOP2A mRNA expression extending it to the adjuvant treatment setting in early high-risk breast cancer. HER2 and TOP2A amplification do not share the same effect on their downstream molecules, with consistent patterns of HER2 mRNA and protein expression according to HER2 amplification, but inconsistent patterns in the case of TOP2A. We are in the process of validating these findings in a larger phase III study with more than 1,000 randomized patients. The strong adverse prognostic impact of high HER2/TOP2A mRNA co-expression needs further validation in studies designed to evaluate markers predictive of response to anthracyclines.

## Competing interests

On behalf of the Hellenic Foundation for Cancer Research, Athens, Greece, the senior investigator author (GF) has pending patent applications with Siemens Healthcare Diagnostics, Tarrytown, NY, USA.

The senior investigator (GF) has received Commercial Research Funding by Roche Hellas SA and Genesis Pharma SA, Athens, Greece.

## Authors' contributions

GF conceived the study, participated in its design, in the collection and assembly of data, in data analysis and interpretation, provided study materials/patients and contributed in the process of manuscript writing, CV conceived the study, participated in its design, in the collection and assembly of data, in data analysis and interpretation and contributed in the process of manuscript writing, VK conceived the study, participated in its design, in the collection and assembly of data, in data analysis and interpretation, and contributed in the process of manuscript writing, AGE participated in data analysis and interpretation and manuscript writing, KTK participated in data analysis and interpretation and manuscript writing, OT conceived the study, participated in its design, in the collection and assembly of data, in data analysis and interpretation and contributed in the process of manuscript writing, AB participated in data analysis and interpretation and manuscript writing, RK participated in the collection and assembly of data and contributed in the process of manuscript writing, RMW participated in the collection and assembly of data and contributed in the process of manuscript writing, MB participated in the collection and assembly of data, in data analysis and interpretation, provided study materials/patients and contributed in the process of manuscript writing, ET participated in the collection and assembly of data and provided study materials/patients, NS participated in the collection and assembly of data and provided study materials/patients, GP participated in the collection and assembly of data and provided study materials/patients, HG participated in the collection and assembly of data, provided study materials/patients and contributed in the process of manuscript writing, DV participated in the collection and assembly of data, in data analysis and interpretation and contributed in the process of manuscript writing, GP participated in the collection and assembly of data, GA participated in the collection and assembly of data and provided study materials/patients, AK participated in the collection and assembly of data, provided study materials/patients and contributed in the process of manuscript writing, CC participated in the collection and assembly of data, provided study materials/patients and contributed in the process of manuscript writing, DP participated in the collection and assembly of data, provided study materials/patients and contributed in the process of manuscript writing, PA conceived the study, participated in its design, in data analysis and interpretation and contributed in the process of manuscript writing. All the authors read and approved the final manuscript.

## References

[B1] Effects of chemotherapy and hormonal therapy for early breast cancer on recurrence and 15-year survival: an overview of the randomised trials Lancet 2005 365 1687 1717 15894097 10.1016/S0140-6736(05)66544-0

[B2] Smith I Chua S Medical treatment of early breast cancer. III: chemotherapy BMJ (Clinical research ed 2006 332 161 162 10.1136/bmj.332.7534.161 PMC1336770 16424494

[B3] Doyle JJ Neugut AI Jacobson JS Grann VR Hershman DL Chemotherapy and cardiotoxicity in older breast cancer patients: a population-based study J Clin Oncol 2005 23 8597 8605 10.1200/JCO.2005.02.5841 16314622

[B4] Hershman D Neugut AI Jacobson JS Wang J Tsai WY McBride R Bennett CL Grann VR Acute myeloid leukemia or myelodysplastic syndrome following use of granulocyte colony-stimulating factors during breast cancer adjuvant chemotherapy Journal of the National Cancer Institute 2007 99 196 205 10.1093/jnci/djk028 17284714

[B5] Ross JS Slodkowska EA Symmans WF Pusztai L Ravdin PM Hortobagyi GN The HER-2 receptor and breast cancer: ten years of targeted anti-HER-2 therapy and personalized medicine The oncologist 2009 14 320 368 10.1634/theoncologist.2008-0230 19346299

[B6] Slamon DJ Clark GM Wong SG Levin WJ Ullrich A McGuire WL Human breast cancer: correlation of relapse and survival with amplification of the HER-2/neu oncogene Science (New York, NY 1987 235 177 182 10.1126/science.3798106 3798106

[B7] Jarvinen TA Liu ET Simultaneous amplification of HER-2 (ERBB2) and topoisomerase IIalpha (TOP2A) genes--molecular basis for combination chemotherapy in cancer Current cancer drug targets 2006 6 579 602 10.2174/156800906778742497 17100565

[B8] Hayes DF Thor AD Dressler LG Weaver D Edgerton S Cowan D Broadwater G Goldstein LJ Martino S Ingle JN HER2 and response to paclitaxel in node-positive breast cancer The New England journal of medicine 2007 357 1496 1506 10.1056/NEJMoa071167 17928597

[B9] Gennari A Sormani MP Pronzato P Puntoni M Colozza M Pfeffer U Bruzzi P HER2 status and efficacy of adjuvant anthracyclines in early breast cancer: a pooled analysis of randomized trials Journal of the National Cancer Institute 2008 100 14 20 10.1093/jnci/djm252 18159072

[B10] Di Leo A Larsimont D Gancberg D Jarvinen T Beauduin M Vindevoghel A Michel J Focan CH Ries F Gobert PH HER-2 and topo-isomerase IIalpha as predictive markers in a population of node-positive breast cancer patients randomly treated with adjuvant CMF or epirubicin plus cyclophosphamide Ann Oncol 2001 12 1081 1089 10.1023/A:1011669223035 11583189

[B11] Konecny GE Thomssen C Luck HJ Untch M Wang HJ Kuhn W Eidtmann H du Bois A Olbricht S Steinfeld D Her-2/neu gene amplification and response to paclitaxel in patients with metastatic breast cancer Journal of the National Cancer Institute 2004 96 1141 1151 10.1093/jnci/djh198 15292386

[B12] Champoux JJ DNA topoisomerases: structure, function, and mechanism Annual review of biochemistry 2001 70 369 413 10.1146/annurev.biochem.70.1.369 11395412

[B13] Ju BG Lunyak VV Perissi V Garcia-Bassets I Rose DW Glass CK Rosenfeld MG A topoisomerase IIbeta-mediated dsDNA break required for regulated transcription Science (New York, NY 2006 312 1798 1802 10.1126/science.1127196 16794079

[B14] Hicks DG Tubbs RR Assessment of the HER2 status in breast cancer by fluorescence in situ hybridization: a technical review with interpretive guidelines Human pathology 2005 36 250 261 10.1016/j.humpath.2004.11.010 15791569

[B15] Schindlbeck C Janni W Shabani N Kornmeier A Rack B Rjosk D Gerber B Braun S Sommer H Friese K Isolated tumor cells in the bone marrow (ITC-BM) of breast cancer patients before and after anthracyclin based therapy: influenced by the HER2- and Topoisomerase IIalpha-status of the primary tumor? Journal of cancer research and clinical oncology 2005 131 539 546 10.1007/s00432-005-0683-y 15887027 PMC12161271

[B16] Lambros MB Natrajan R Reis-Filho JS Chromogenic and fluorescent in situ hybridization in breast cancer Human pathology 2007 38 1105 1122 10.1016/j.humpath.2007.04.011 17640550

[B17] Fountzilas G Skarlos D Dafni U Gogas H Briasoulis E Pectasides D Papadimitriou C Markopoulos C Polychronis A Kalofonos HP Postoperative dose-dense sequential chemotherapy with epirubicin, followed by CMF with or without paclitaxel, in patients with high-risk operable breast cancer: a randomized phase III study conducted by the Hellenic Cooperative Oncology Group Ann Oncol 2005 16 1762 1771 10.1093/annonc/mdi366 16148021

[B18] Pritchard KI Are HER2 and TOP2A useful as prognostic or predictive biomarkers for anthracycline-based adjuvant chemotherapy for breast cancer? J Clin Oncol 2009 27 3875 3876 10.1200/JCO.2009.22.8361 19620479

[B19] Kononen J Bubendorf L Kallioniemi A Barlund M Schraml P Leighton S Torhorst J Mihatsch MJ Sauter G Kallioniemi OP Tissue microarrays for high-throughput molecular profiling of tumor specimens Nature medicine 1998 4 844 847 10.1038/nm0798-844 9662379

[B20] Skacel M Skilton B Pettay JD Tubbs RR Tissue microarrays: a powerful tool for high-throughput analysis of clinical specimens: a review of the method with validation data Appl Immunohistochem Mol Morphol 2002 10 1 6 10.1097/00022744-200203000-00001 11893029

[B21] Christodoulou C Kostopoulos I Kalofonos HP Lianos E Bobos M Briasoulis E Gogas H Razis E Skarlos DV Fountzilas G Trastuzumab combined with pegylated liposomal doxorubicin in patients with metastatic breast cancer. phase II Study of the Hellenic Cooperative Oncology Group (HeCOG) with biomarker evaluation Oncology 2009 76 275 285 10.1159/000207504 19262067

[B22] Tanner M Gancberg D Di Leo A Larsimont D Rouas G Piccart MJ Isola J Chromogenic in situ hybridization: a practical alternative for fluorescence in situ hybridization to detect HER-2/neu oncogene amplification in archival breast cancer samples The American journal of pathology 2000 157 1467 1472 10.1016/S0002-9440(10)64785-2 11073807 PMC1885742

[B23] Wolff AC Hammond ME Schwartz JN Hagerty KL Allred DC Cote RJ Dowsett M Fitzgibbons PL Hanna WM Langer A American Society of Clinical Oncology/College of American Pathologists guideline recommendations for human epidermal growth factor receptor 2 testing in breast cancer Archives of pathology & laboratory medicine 2007 131 18 43 19548375 10.5858/2007-131-18-ASOCCO

[B24] Bohmann K Hennig G Rogel U Poremba C Mueller BM Fritz P Stoerkel S Schaefer KL RNA extraction from archival formalin-fixed paraffin-embedded tissue: a comparison of manual, semiautomated, and fully automated purification methods Clin Chem 2009 55 1719 1727 10.1373/clinchem.2008.122572 19617290

[B25] Hennig G Gehrmann M Stropp U Brauch H Fritz P Eichelbaum M Schwab M Schroth W Automated extraction of DNA and RNA from a single formalin-fixed paraffin-embedded tissue section for analysis of both single-nucleotide polymorphisms and mRNA expression Clinical chemistry 2010 56 1845 1853 10.1373/clinchem.2010.151233 20947696

[B26] Muller BM Kronenwett R Hennig G Euting H Weber K Bohmann K Weichert W Altmann G Roth C Winzer KJ Quantitative determination of estrogen receptor, progesterone receptor, and HER2 mRNA in formalin-fixed paraffin-embedded tissue--a new option for predictive biomarker assessment in breast cancer Diagn Mol Pathol 2011 20 1 10 10.1097/PDM.0b013e3181e3630c 21326033

[B27] Bhargava R Lal P Chen B HER-2/neu and topoisomerase IIa gene amplification and protein expression in invasive breast carcinomas: chromogenic in situ hybridization and immunohistochemical analyses American journal of clinical pathology 2005 123 889 895 10.1309/PCFK8YTQPYWD534F 15899781

[B28] Cheang MC Chia SK Voduc D Gao D Leung S Snider J Watson M Davies S Bernard PS Parker JS Ki67 index, HER2 status, and prognosis of patients with luminal B breast cancer Journal of the National Cancer Institute 2009 101 736 750 10.1093/jnci/djp082 19436038 PMC2684553

[B29] Hammond ME Hayes DF Dowsett M Allred DC Hagerty KL Badve S Fitzgibbons PL Francis G Goldstein NS Hayes M American Society of Clinical Oncology/College Of American Pathologists guideline recommendations for immunohistochemical testing of estrogen and progesterone receptors in breast cancer J Clin Oncol 2010 28 2784 2795 10.1200/JCO.2009.25.6529 20404251 PMC2881855

[B30] Hudis CA Barlow WE Costantino JP Gray RJ Pritchard KI Chapman JA Sparano JA Hunsberger S Enos RA Gelber RD Zujewski JA Proposal for standardized definitions for efficacy end points in adjuvant breast cancer trials: the STEEP system J Clin Oncol 2007 25 2127 2132 10.1200/JCO.2006.10.3523 17513820

[B31] McShane LM Altman DG Sauerbrei W Taube SE Gion M Clark GM REporting recommendations for tumor MARKer prognostic studies (REMARK) Breast cancer research and treatment 2006 100 229 235 10.1007/s10549-006-9242-8 16932852

[B32] Simon RM Paik S Hayes DF Use of archived specimens in evaluation of prognostic and predictive biomarkers Journal of the National Cancer Institute 2009 101 1446 1452 10.1093/jnci/djp335 19815849 PMC2782246

[B33] Nielsen KV Muller S Moller S Schonau A Balslev E Knoop AS Ejlertsen B Aberrations of ERBB2 and TOP2A genes in breast cancer Molecular oncology 2010 4 161 168 10.1016/j.molonc.2009.11.001 19945923 PMC5527893

[B34] De P Smith BR Leyland-Jones B Human epidermal growth factor receptor 2 testing: where are we? J Clin Oncol 2010 28 4289 4292 10.1200/JCO.2010.29.5071 20697080

[B35] Shah SS Ketterling RP Goetz MP Ingle JN Reynolds CA Perez EA Chen B Impact of American Society of Clinical Oncology/College of American Pathologists guideline recommendations on HER2 interpretation in breast cancer Human pathology 2010 41 103 106 10.1016/j.humpath.2009.07.001 19762065

[B36] Brase JC Schmidt M Fischbach T Sultmann H Bojar H Koelbl H Hellwig B Rahnenfuhrer J Hengstler JG Gehrmann MC ERBB2 and TOP2A in breast cancer: a comprehensive analysis of gene amplification, RNA levels, and protein expression and their influence on prognosis and prediction Clin Cancer Res 2010 16 2391 2401 10.1158/1078-0432.CCR-09-2471 20371687

[B37] van de Vijver M Bilous M Hanna W Hofmann M Kristel P Penault-Llorca F Ruschoff J Chromogenic in situ hybridisation for the assessment of HER2 status in breast cancer: an international validation ring study Breast Cancer Res 2007 9 R68 10.1186/bcr1776 17922920 PMC2242665

[B38] Moelans CB de Weger RA van Blokland MT van der Wall E van Diest PJ Simultaneous detection of TOP2A and HER2 gene amplification by multiplex ligation-dependent probe amplification in breast cancer Mod Pathol 2010 23 62 70 10.1038/modpathol.2009.136 19767729

[B39] Bartlett JM Munro AF Dunn JA McConkey C Jordan S Twelves CJ Cameron DA Thomas J Campbell FM Rea DW Predictive markers of anthracycline benefit: a prospectively planned analysis of the UK National Epirubicin Adjuvant Trial (NEAT/BR9601) The lancet oncology 2010 11 266 274 10.1016/S1470-2045(10)70006-1 20079691

[B40] Jarvinen TA Tanner M Barlund M Borg A Isola J Characterization of topoisomerase II alpha gene amplification and deletion in breast cancer Genes, chromosomes & cancer 1999 26 142 150 10.1002/(SICI)1098-2264(199910)26:2<142::AID-GCC6>3.0.CO;2-B 10469452

[B41] Usha L Tabesh B Morrison LE Rao RD Jacobson K Zhu A Basu S Coon JS Topoisomerase II alpha gene copy loss has adverse prognostic significance in ERBB2-amplified breast cancer: a retrospective study of paraffin-embedded tumor specimens and medical charts Journal of hematology & oncology 2008 1 12 10.1186/1756-8722-1-12 18702822 PMC2546432

[B42] Press MF Sauter G Buyse M Bernstein L Guzman R Santiago A Villalobos IE Eiermann W Pienkowski T Martin M Alteration of topoisomerase II-alpha gene in human breast cancer: association with responsiveness to anthracycline-based chemotherapy J Clin Oncol 2011 29 859 867 10.1200/JCO.2009.27.5644 21189395 PMC3068060

[B43] Park K Kim J Lim S Han S Topoisomerase II-alpha (topoII) and HER2 amplification in breast cancers and response to preoperative doxorubicin chemotherapy Eur J Cancer 2003 39 631 634 10.1016/S0959-8049(02)00745-1 12628842

[B44] O'Malley FP Chia S Tu D Shepherd LE Levine MN Bramwell VH Andrulis IL Pritchard KI Topoisomerase II alpha and responsiveness of breast cancer to adjuvant chemotherapy Journal of the National Cancer Institute 2009 101 644 650 10.1093/jnci/djp067 19401546 PMC2677575

[B45] Ejlertsen B Jensen MB Nielsen KV Balslev E Rasmussen BB Willemoe GL Hertel PB Knoop AS Mouridsen HT Brunner N HER2, TOP2A, and TIMP-1 and responsiveness to adjuvant anthracycline-containing chemotherapy in high-risk breast cancer patients J Clin Oncol 2010 28 984 990 10.1200/JCO.2009.24.1166 20038724

[B46] Desmedt C Di Leo A de Azambuja E Larsimont D Haibe-Kains B Selleslags J Delaloge S Duhem C Kains JP Carly B Multifactorial approach to predicting resistance to anthracyclines J Clin Oncol 2011 29 1578 1586 10.1200/JCO.2010.31.2231 21422418

[B47] Yeh IT Martin MA Robetorye RS Bolla AR McCaskill C Shah RK Gorre ME Mohammed MS Gunn SR Clinical validation of an array CGH test for HER2 status in breast cancer reveals that polysomy 17 is a rare event Mod Pathol 2009 22 1169 1175 10.1038/modpathol.2009.78 19448591

[B48] Moelans CB de Weger RA Van der Wall E van Diest PJ Current technologies for HER2 testing in breast cancer Critical reviews in oncology/hematology 2011 10.1016/j.critrevonc.2010.12.005 21273092

[B49] Coon JS Marcus E Gupta-Burt S Seelig S Jacobson K Chen S Renta V Fronda G Preisler HD Amplification and overexpression of topoisomerase IIalpha predict response to anthracycline-based therapy in locally advanced breast cancer Clin Cancer Res 2002 8 1061 1067 11948114

[B50] Mueller RE Parkes RK Andrulis I O'Malley FP Amplification of the TOP2A gene does not predict high levels of topoisomerase II alpha protein in human breast tumor samples Genes, chromosomes & cancer 2004 39 288 297 10.1002/gcc.20008 14978790

[B51] Romero A Martin M Cheang MC Lopez Garcia-Asenjo JA Oliva B He X de la Hoya M Garcia Saenz JA Arroyo Fernandez M Diaz Rubio E Assessment of Topoisomerase II alpha status in breast cancer by quantitative PCR, gene expression microarrays, immunohistochemistry, and fluorescence in situ hybridization The American journal of pathology 2011 178 1453 1460 10.1016/j.ajpath.2010.12.042 21435434 PMC3078442

[B52] Sandri MI Hochhauser D Ayton P Camplejohn RC Whitehouse R Turley H Gatter K Hickson ID Harris AL Differential expression of the topoisomerase II alpha and beta genes in human breast cancers British journal of cancer 1996 73 1518 1524 10.1038/bjc.1996.286 8664122 PMC2074549

[B53] Stacey DW Hitomi M Chen G Influence of cell cycle and oncogene activity upon topoisomerase IIalpha expression and drug toxicity Molecular and cellular biology 2000 20 9127 9137 10.1128/MCB.20.24.9127-9137.2000 11094065 PMC102171

[B54] Sandri MI Isaacs RJ Ongkeko WM Harris AL Hickson ID Broggini M Vikhanskaya F p53 regulates the minimal promoter of the human topoisomerase IIalpha gene Nucleic acids research 1996 24 4464 4470 10.1093/nar/24.22.4464 8948636 PMC146283

[B55] Stros M Polanska E Struncova S Pospisilova S HMGB1 and HMGB2 proteins up-regulate cellular expression of human topoisomerase IIalpha Nucleic acids research 2009 37 2070 2086 10.1093/nar/gkp067 19223331 PMC2673423

[B56] Goswami PC Sheren J Albee LD Parsian A Sim JE Ridnour LA Higashikubo R Gius D Hunt CR Spitz DR Cell cycle-coupled variation in topoisomerase IIalpha mRNA is regulated by the 3'-untranslated region. Possible role of redox-sensitive protein binding in mRNA accumulation The Journal of biological chemistry 2000 275 38384 38392 10986283 10.1074/jbc.M005298200

[B57] Beresford MJ Wilson GD Makris A Measuring proliferation in breast cancer: practicalities and applications Breast Cancer Res 2006 8 216 10.1186/bcr1618 17164010 PMC1797032

[B58] Nitiss JL DNA topoisomerase II and its growing repertoire of biological functions Nature reviews 2009 9 327 337 10.1038/nrc2608 19377505 PMC2730144

[B59] Arriola E Moreno A Varela M Serra JM Falo C Benito E Escobedo AP Predictive value of HER-2 and Topoisomerase IIalpha in response to primary doxorubicin in breast cancer Eur J Cancer 2006 42 2954 2960 10.1016/j.ejca.2006.06.013 16935488

[B60] Yerushalmi R Woods R Ravdin PM Hayes MM Gelmon KA Ki67 in breast cancer: prognostic and predictive potential The lancet oncology 2010 11 174 183 10.1016/S1470-2045(09)70262-1 20152769

[B61] Rody A Karn T Ruckhaberle E Muller V Gehrmann M Solbach C Ahr A Gatje R Holtrich U Kaufmann M Gene expression of topoisomerase II alpha (TOP2A) by microarray analysis is highly prognostic in estrogen receptor (ER) positive breast cancer Breast cancer research and treatment 2009 113 457 466 10.1007/s10549-008-9964-x 18340528

[B62] Cardoso F Durbecq V Larsimont D Paesmans M Leroy JY Rouas G Sotiriou C Renard N Richard V Piccart MJ Di Leo A Correlation between complete response to anthracycline-based chemotherapy and topoisomerase II-alpha gene amplification and protein overexpression in locally advanced/metastatic breast cancer International journal of oncology 2004 24 201 209 14654958

[B63] Nitiss JL Targeting DNA topoisomerase II in cancer chemotherapy Nature reviews 2009 9 338 350 19377506 10.1038/nrc2607 PMC2748742

[B64] Knoop AS Knudsen H Balslev E Rasmussen BB Overgaard J Nielsen KV Schonau A Gunnarsdottir K Olsen KE Mouridsen H Ejlertsen B retrospective analysis of topoisomerase IIa amplifications and deletions as predictive markers in primary breast cancer patients randomly assigned to cyclophosphamide, methotrexate, and fluorouracil or cyclophosphamide, epirubicin, and fluorouracil: Danish Breast Cancer Cooperative Group J Clin Oncol 2005 23 7483 7490 10.1200/JCO.2005.11.007 16234514

[B65] Natrajan R Lambros MB Rodriguez-Pinilla SM Moreno-Bueno G Tan DS Marchio C Vatcheva R Rayter S Mahler-Araujo B Fulford LG Tiling path genomic profiling of grade 3 invasive ductal breast cancers Clin Cancer Res 2009 15 2711 2722 10.1158/1078-0432.CCR-08-1878 19318498

[B66] Rody A Karn T Gatje R Ahr A Solbach C Kourtis K Munnes M Loibl S Kissler S Ruckhaberle E Gene expression profiling of breast cancer patients treated with docetaxel, doxorubicin, and cyclophosphamide within the GEPARTRIO trial: HER-2, but not topoisomerase II alpha and microtubule-associated protein tau, is highly predictive of tumor response Breast (Edinburgh, Scotland) 2007 16 86 93 10.1016/j.breast.2006.06.008 17010609

[B67] Koutras AK Kalogeras KT Dimopoulos MA Wirtz RM Dafni U Briasoulis E Pectasides D Gogas H Christodoulou C Aravantinos G Evaluation of the prognostic and predictive value of HER family mRNA expression in high-risk early breast cancer: a Hellenic Cooperative Oncology Group (HeCOG) study British journal of cancer 2008 99 1775 1785 10.1038/sj.bjc.6604769 18985033 PMC2600696

[B68] Pusztai L Jeong JH Gong Y Ross JS Kim C Paik S Rouzier R Andre F Hortobagyi GN Wolmark N Symmans WF Evaluation of microtubule-associated protein-Tau expression as a prognostic and predictive marker in the NSABP-B 28 randomized clinical trial Journal of clinical oncology: official journal of the American Society of Clinical Oncology 2009 27 4287 4292 10.1200/JCO.2008.21.6887 PMC2744271 19667268

[B69] Dumontet C Krajewska M Treilleux I Mackey JR Martin M Rupin M Lafanechere L Reed JC BCIRG 001 molecular analysis: prognostic factors in node-positive breast cancer patients receiving adjuvant chemotherapy Clinical cancer research: an official journal of the American Association for Cancer Research 2010 16 3988 3997 10.1158/1078-0432.CCR-10-0079 20576719

[B70] Noske A Loibl S Darb-Esfahani S Roller M Kronenwett R Muller BM Steffen J von Toerne C Wirtz R Baumann I Comparison of different approaches for assessment of HER2 expression on protein and mRNA level: prediction of chemotherapy response in the neoadjuvant GeparTrio trial (NCT00544765) Breast cancer research and treatment 2011 126 109 117 10.1007/s10549-010-1316-y 21190079

[B71] Bergqvist J Ohd JF Smeds J Klaar S Isola J Nordgren H Elmberger GP Hellborg H Bjohle J Borg AL Quantitative real-time PCR analysis and microarray-based RNA expression of HER2 in relation to outcome Ann Oncol 2007 18 845 850 10.1093/annonc/mdm059 17351254

[B72] Barberis M Pellegrini C Cannone M Arizzi C Coggi G Bosari S Quantitative PCR and HER2 testing in breast cancer: a technical and cost-effectiveness analysis American journal of clinical pathology 2008 129 563 570 10.1309/1AKQDQ057PQT9AKX 18343783

[B73] Cronin M Sangli C Liu ML Pho M Dutta D Nguyen A Jeong J Wu J Langone KC Watson D Analytical validation of the Oncotype DX genomic diagnostic test for recurrence prognosis and therapeutic response prediction in node-negative, estrogen receptor-positive breast cancer Clinical chemistry 2007 53 1084 1091 10.1373/clinchem.2006.076497 17463177

